# Evaluation du risque thromboembolique veineux et pratique de la thromboprophylaxie en médecine interne

**DOI:** 10.11604/pamj.2015.22.386.7988

**Published:** 2015-12-29

**Authors:** Diatou Gueye Dia, Seynabou Fall, Amadou Diop Dia, Nafissatou Diagne Sakho, Sidy Mohamed Seck, Thérèse Moreira Diop

**Affiliations:** 1 UFR des Sciences de la Santé, Université Gaston Berger, Saint-Louis, Sénégal; 2 Clinique Médicale 1 CHU Aristide le Dantec, Dakar, Sénégal

**Keywords:** Maladie thrombo-embolique, thromboprophylaxie, héparine, thromboembolic disease, thromboprophylaxis, heparin

## Abstract

**Introduction:**

Le risque thromboembolique veineux en médecine a été largement incriminé dans la charge humaine et financière de l'ensemble de cette pathologie. Les facteurs de risque sont identifiés et côtés pour optimiser la prise en charge. Notre objectif était d’évaluer le niveau de risque thromboembolique et la pratique de la thromboprophylaxie.

**Méthodes:**

Il s'agissait d'une étude rétrospective réalisée sur une durée de 12 mois dans le service de médecine interne du CHU le Dantec. L'inclusion des patients était systématique à l'exclusion des patients ayant une durée d'hospitalisation de moins de 3 jours et de ceux venus avec un traitement anticoagulant.

**Résultats:**

Nous avons colligé 352 dossiers. Le sexe ratio était à 1,21 en faveur des hommes. L’âge moyen des patients est de 47ans. Le niveau de risque thromboembolique a été faible dans 23%, modéré dans 22%, élevé dans 36%, et maximal dans 19% des cas. Les facteurs de risque retrouvés sont représentés par l'alitement (98%), l’âge compris entre 41-74 ans (47%), les néoplasies (20,1%). L'insuffisance cardiaque et les affections respiratoires graves sont rapportées chez respectivement 9,3% et 8,5% des patients. Une prophylaxie était nécessaire chez 77% des patients hospitalisés mais seuls 12% des patients en avait bénéficié.

**Conclusion:**

La nécessité d'une prévention de la maladie thromboembolique veineuse est bien cernée par les praticiens mais se heurte à de nombreux obstacles d'où la nécessité d'une mise en place d'outils pratiques et fonctionnels de dépistages et de produits anticoagulants accessibles.

## Introduction

La maladie veineuse thromboembolique regroupe la thrombose veineuse profonde et son risque immédiat l'embolie pulmonaire. Depuis l’énoncé en 1860 par Virchow de la triade physiopathologique sous-tendant la MTEV, l’épidémiologie clinique a contribué à améliorer la connaissance de cette pathologie en identifiant ses facteurs de risque [1]. Avec l’évolution des connaissances médicales les causes de lésions vasculaires endothéliales se sont révélées être diversifiées et impliquent de plus en plus les phénomènes inflammatoires et/ ou infectieux, les phénomènes athéromateux et oncologiques [1, 2]. Les différentes réunions de consensus de l'American College of Chest Physicians (ACCP), n'ont cessé de tirer la sonnette d'alarme en direction des affections médicales: 50 à 70% des événements thromboemboliques symptomatiques surviennent en milieu hospitalier chez des patients issus de services médicaux [3]. Au Sénégal, et en Afrique de manière plus générale, la plupart des travaux ont été menés dans les services d'anatomie pathologique et de cardiologie [4, 5]. Cette étude a été menée avec comme objectifs: d'identifier les facteurs de risque thromboembolique veineux et de déterminer le niveau de risque thromboembolique veineux ainsi que la prise en charge prophylactique.

## Méthodes

Il s'agissait d'une étude descriptive rétrospective sur une période de 12 mois. Elle porte sur l'analyse de 352 dossiers de patients admis dans le service de médecine interne de l'Hôpital Aristide Le Dantec. Durant la période de l’étude tous les patients hospitalisés depuis au moins 3 jours ont été inclus quelque soit l’âge et le sexe. Les dossiers ont été dépouillés à l'aide d'un formulaire inspiré du score de Caprini [6]. Les facteurs de risque ont été recherchés à partir du diagnostic principal; du diagnostic associé mais aussi à partir de l'exploitation de chaque dossier depuis l'interrogatoire jusqu'aux différentes étapes de la prise en charge des patients. A chaque facteur de risque il a été attribué un score et le score total permet de définir le niveau de risque. Le risque est faible pour un total à 0 ou 1, modéré par un total à 2, élevé pour un total à 3 ou 4 et maximal au-delà de 5. A chaque niveau de risque est corrélée une prophylaxie adaptée. Ces éléments avaient permis d'une part d’évaluer le niveau de risque thromboembolique et d'autre part la prophylaxie adaptée en accord avec les recommandations de l'ACCP. Les critères de non inclusion étaient une durée d'hospitalisation de moins de 3 jours et un traitement anticoagulant antérieur à l'admission. L'analyse statistique a été faite à l'aide du logiciel Epi info 6. Des analyses univariées ont été faites pour le calcul des fréquences, des moyennes et des écart-types. Les analyses bi variées avaient permis de rechercher les corrélations entre les différents variables. Le test utilisé pour les comparaisons était le test de khi-2 lorsque l'effectif était supérieur ou égal à 5 dans toutes les classes. Lorsque l'effectif était inférieur à 5 dans une classe, c'est le test de Fisher qui a été utilisé. Le résultat est statistiquement significatif si le p est inférieur à 0,05.

## Résultats

**Figure 1:**
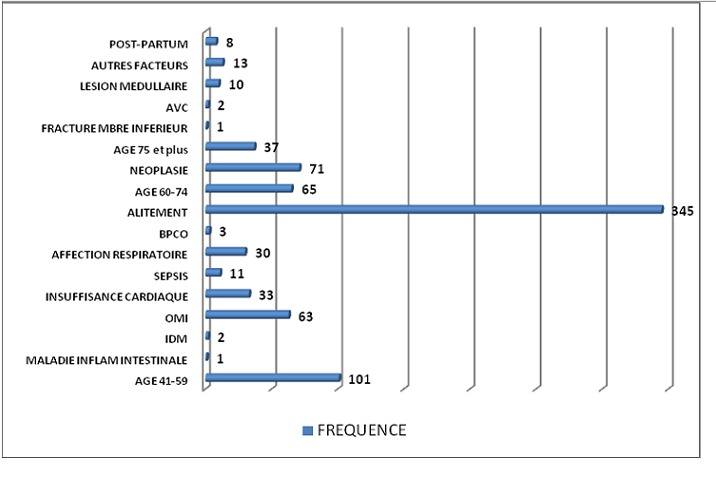
Effectif des différents facteurs de risque

**Figure 2:**
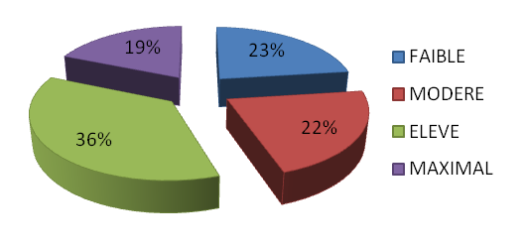
Répartition du niveau de risque

L’âge moyen des patients était 47 ± 19,20 ans avec des extrêmes entre 16 ans et 99 ans. La population se répartissait en 159 femmes soit 45% et 193 hommes soit 55% avec un sexe ratio à 1,21. La durée moyenne d'hospitalisation était de 16 jours avec des extrêmes allant de 3 à 124 jours. Les facteurs de risque thromboembolique (Figure 1) sont représentés surtout par l'alitement (98%), la tranche d’âge 41-74 ans (47%) et les néoplasies (20,1%). L'insuffisance cardiaque et les affections respiratoires graves étaient retrouvées respectivement chez 9,3% et 8,5% des patients. Nous n'avons pas retrouvé de cas de thrombophilie. Le niveau de risque thromboembolique a été stratifié en quatre degrés: il a été faible chez 81 patients (23%), modéré chez 77 patients (22%), élevé chez 128 patients (36%) et maximal chez 66 patients (19%). Il est représenté par la Figure 2. L'attitude préventive de la maladie thromboembolique veineuse a été correcte chez 112 patients soit 32,55%. Parmi les 263 patients (soit 77% des malades hospitalisés) qui nécessitaient une prophylaxie, seuls 31 l'ont effectivement reçu soit 12%. Lorsque le risque était est modéré, la prévention n'a été entreprise que dans 6% des cas. Elle était est de 14,6% lorsque le risque était élevé et de 20% si le risque était est maximal. Le Tableau 1 représente le pourcentage de prévention en fonction du niveau de risque.

**Tableau 1:** Pourcentage de prévention en fonction du niveau de risque

Prévention	Oui n (%)	Non n (%)	Total
**Niveau de risque**			
Modéré	4(6)	68(94)	72
Elevé	16 (14,6)	109(85,4)	125
Maximal	11(20)	55(80)	66

## Discussion

Ce travail montre que 77% des patients présentent un risque de MTEV requérant une prophylaxie. L’étude Endorse retrouvait en milieu médical que le pourcentage de malades hospitalisés nécessitant une prophylaxie était de 57,4% au Sénégal [7] et 46,5% en Tunisie [8]. Dans notre étude, le niveau de risque était faible modéré à élevé chez plus de la moitié des patients (58%). Ces constats rejoignent ceux de l’étude de Dèdonougbo^9^ qui observait un risque faible dans 12,9%, modéré dans 42%% et élevé dans 45,1%. Les facteurs de risque thromboembolique que nous avons retrouvés dans cette étude étaient représentés surtout par l'alitement (98%), l’âge supérieur à 40 ans (47%) et les néoplasies (20,1%). L'insuffisance cardiaque était présente dans 9,3% des cas et les affections respiratoires graves dans 8,5% des cas. Dans les résultats d'Endorse en Tunisie [8], ces facteurs de risque étaient dominés par les pathologies cardio-vasculaires à 40% suivies de l'infection pulmonaire à 36,1%. Poittier [10] retrouvait dans sa série une prépondérance de facteurs de stase avec l’âge supérieur à 60 ans à 70%, l'insuffisance cardiaque à 17%, un cancer évolutif à 8%. Concernant les mesures préventives; parmi les 77% des patients qui nécessitaient une prophylaxie, seuls 12% en avaient bénéficié. De nombreux travaux se sont interréssés récemment à la prévention de la MTEV en milieu médical mais les résultats sont variables. Dans la série de Dèdonougbo [9], la prévention n'a été appropriée que dans 6% des cas. Dans une étude faite en 2006 dans la ville de Manaus au Brésil par Lee [11] portant sur 1036 patients hospitalisés, la prophylaxie n'a été faite que chez 26% des patients présentant un niveau de risque modéré ou élevé. Cette prévention était de 33% en Afrique du Nord [12]. Globalement les taux de prévention les plus élevés sont obtenus dans les pays du Nord. Elle est de 51,8% en Europe de l'Ouest et aux USA avec une moyenne mondiale de 48% [12]. Sur le plan thérapeutique, seules les héparines de bas poids moléculaire ont été utilisées dans cette série. Poittier [13] retrouvait dans un service de médecine interne que 42% des patients avait bénéficié d'un traitement préventif par héparines de bas poids moléculaire dont 38% à juste titre (selon les critères proposés) et 4% par excès. Dans ce travail, la fréquence globale des contre-indications à l'anticoagulation était de 21,3%. Dans les résultats focaux de Endorse Sénégal [7], cette fréquence était est de 7,9% en médecine et chez ces patients la contre-indication n'a pas été respectée dans 3,4% contre 14,6% dans notre série. Les contre-indications étaient dans cette étude dominées par les anomalies de la clairance de la créatinine (41%), suivies des thrombopénies (31%) puis la présence d'un saignenent actif (25%) et les traitements antiagrégants (3%). Par contre dans l’étude Endorse Internationationale [12], les thrombopénies étaient présentes à 4% et les perturbations rénales à 11%. Ceci pourrait s'expliquer par la fréquence des pathologies rénales et hématologiques du fait que cette étude a été réalisée dans un service de médecine interne.

## Conclusion

Cette étude montre qu’à travers les facteurs de risque qui sont actuellement bien définis, plus de 77% des patients devraient bénéficier d'une thromboprophylaxie. Des efforts doivent encore être faits dans la prévention de cette pathologie. Ce qui souligne la nécessité de faire un plaidoyer auprès des instances politiques pour une accéssibilité en terme de disponibilité continue et de réduction des prix des molécules.
